# Work in nursing homes and occupational exposure to endotoxin and bacterial and fungal species

**DOI:** 10.1093/annweh/wxad032

**Published:** 2023-06-10

**Authors:** Pil Uthaug Rasmussen, Katrine Uhrbrand, Margit W Frederiksen, Anne Mette Madsen

**Affiliations:** National Research Centre for the Working Environment, Lersø Parkallé 105, 2100 Copenhagen Ø, Denmark; National Research Centre for the Working Environment, Lersø Parkallé 105, 2100 Copenhagen Ø, Denmark; National Research Centre for the Working Environment, Lersø Parkallé 105, 2100 Copenhagen Ø, Denmark; National Research Centre for the Working Environment, Lersø Parkallé 105, 2100 Copenhagen Ø, Denmark

**Keywords:** antibiotic resistance, azole, bioaerosols, healthcare worker (HCW), long-term care facility (LTCF), occupational health

## Abstract

Indoor microbial exposure may cause negative health effects. Only little is known about the occupational microbial exposure in nursing homes and the factors that influence the exposure. The exposure in nursing homes may be increased due to close contact with elderly persons who may carry infectious or antimicrobial-resistant microorganisms and due to handling of laundry, such as used clothing and bed linen. We investigated the microbial exposure in 5 nursing homes in Denmark, by use of personal bioaerosol samples from different groups of staff members taken during a typical working day, stationary bioaerosol measurements taken during various work tasks, sedimented dust samples, environmental surface swabs, and swabs from staff members’ hands. From the samples, we explored bacterial and fungal concentrations and species composition, endotoxin levels, and antimicrobial resistance in *Aspergillus fumigatus* isolates. Microbial concentrations from personal exposure samples differed among professions, and geometric means (GM) were 2,159 cfu/m^3^ (84 to 1.5 × 10^5^) for bacteria incubated on nutrient agar, 1,745 cfu/m^3^ (82 to 2.0 × 10^4^) for bacteria cultivated on a *Staphylococcus* selective agar, and 16 cfu/m^3^ air for potential pathogenic fungi incubated at 37 °C (below detection limit to 257). Bacterial exposures were elevated during bed making. On surfaces, the highest bacterial concentrations were found on bed railings. The majority of bacterial species found were related to the human skin microflora, such as different *Staphylococcus* and *Corynebacterium* species. Endotoxin levels ranged from 0.02 to 59.0 EU/m^3^, with a GM of 1.5 EU/m^3^. Of 40 tested *A. fumigatus* isolates, we found one multiresistant isolate, which was resistant towards both itraconazole and voriconazole, and one isolate resistant towards amphotericin B. In conclusion, we give an overview of the general microbial exposure in nursing homes and show that microbial exposures are higher for staff with more care and nursing tasks compared with administrative staff.

**What’s important about this paper?**

Nursing homes are settings where there are high risks of infection among residents and health care workers. This study found that microbial exposure is highest in the group of health care workers with the most personal care and nursing tasks (social and health care assistants and helpers). Tasks and areas with elevated microbial concentrations include staphylococcal concentrations during bed making, bacteria concentrations in sedimented dust in staff changing rooms, and bacteria concentrations on surfaces in common areas and bed railings in residents’ rooms.

## Introduction

In Denmark, half of the residents in nursing homes have 1 or more chronic diseases, and the residents have an increased use of medicines, more contact with doctors, and admission to hospitals (Sundheds- og Ældreministeriet, 2016). This leads to a risk of the residents being colonized or infected with harmful or antimicrobial-resistant microorganisms (e.g. Garazi et al., 2009), which may pose an occupational risk for the staff working in the nursing homes.

Bacteria and fungi found in indoor air may stem from a range of sources, such as humans, pets, plants, mould in walls, and from outdoor sources (Peat et al., 1998; Miletto and Lindow, 2015; Jürgensen and Madsen, 2016). The concentration and composition are furthermore determined by season, ventilation, human activity, and the number of residents, as well as the frequency of cleaning (Frankel, Bekö, et al., 2012; Miletto and Lindow, 2015). Indoor airborne microbial exposure has been associated with adverse health outcomes, such as headaches, allergies, and respiratory problems, e.g., asthma (Karottki et al., 2014; Cox et al., 2020; Hyytiäinen et al., 2021). Endotoxin from gram-negative bacteria has also been shown to be linked to health problems, such as reduction of lung function (Karottki et al., 2014).

The dispersal of pathogenic and antibiotic resistant microorganisms in nursing homes have often been investigated with a focus on transmission via contaminated fomites and surfaces, with less attention paid to airborne dispersal. However, as exposure may also happen via air, it is important to study both. Furthermore, it is important to study antibiotic resistant microorganisms, such as fungi. For example, the fungi, *Aspergillus fumigatus*, is the most common cause of fungal infections, and these infections are mainly found in persons with a weakened immune system. Furthermore, isolates of this species have shown resistance towards treatment (e.g. Azevedo et al., 2015; Risum et al., 2022). Therefore, examining the presence and potential resistance to antibiotics in this species and whether nursing home staff are exposed is of interest.

Few studies have examined the work-related microbial exposure in nursing homes; however, in order to mitigate potential exposure problems, it is important to identify the factors influencing exposure. The aim of the study was therefore to investigate the occupational microbial exposure for staff members in nursing homes and to investigate the differences in microbial exposure among professions and areas within the nursing homes, in order to identify job functions where staff members have to be aware of the increased exposure and areas where increased hygiene may reduce exposure.

## Methods

### Study design

Sampling was carried out from October 2017 to June 2018 at 5 nursing homes (A–E) in the Capital Region of Denmark. Nursing homes were visited on 2 occasions, 14 days apart. The number of participants per nursing home visit ranged from 4 to 11 (Supplementary Table S1). Fifty-two employees participated, however, as each nursing home was visited twice some participants took part in both sampling visits, thereby ending with a total of 79 personal exposure assessments. The 79 measurements included 8 measurements from nurses, 37 measurements from social and healthcare assistants (referred to as SOSU assistants), 15 measurements from social and healthcare helpers (referred to as SOSU helpers), 7 measurements from cleaning assistants, and 12 measurements from persons in other job functions, such as SOSU trainees and dieticians. The highest sampled profession were SOSU assistants as this is the most common profession of nursing home staff.

### Sampling of microorganisms

To determine the personal exposure to microorganisms, bioaerosols from the inhalation zone of the nursing home staff were sampled using personal air samplers (Gesamtstaubprobenahme sampler [GSP], CIS, BGI Inc., Waltham, MA, USA) mounted with a 37-mm polycarbonate filter (PC, pore size 1.0 µm, Main Manufacturing, Grand Blanc, MI, USA) and a flow rate of 3.5 L/min (*n* = 79) (Aizenberg et al., 2001; Frankel, Timm, et al., 2012). The GSP sampler is designed to collect the inhalable dust fraction of the air by using an intake velocity of 1.25 m/s at the respective flow rate. This corresponds approximately to the inhalation speed of humans. The average sampling period per personal sample was 315 min.

To determine exposure during specific work tasks stationary short-term air sampling was done using GSP samplers with a PC filter (*n* = 32). Measurements were done in both common areas and residents’ rooms. Here, residents’ rooms refer to one resident per nursing home, which had an MRSA infection (see Rasmussen et al., 2020). Samplers were placed at a height of 1.5 m and sampled for 20 min with a flow rate of 3.5 L/min. Short-term air sampling during tasks was also performed by sampling bacteria directly on SaSelect agar (SA; Bio-Rad, Marnes-la-Coquette, France) using a 6-stage Andersen Cascade Impactor (ACI; N6, Thermo Fisher Scientific Inc., Waltham, MA, USA) with a flow rate of 28.3 L/min (*n* = 42). SaSelect agar is a chromogenic medium, which inhibits the majority of microorganisms with the exception of staphylococci. The ACI sampler uses multistage impaction to collect 6 aerosol size fractions directly on agar medium in Petri dishes, with size fractions ranging from above 7 to 0.65 µm. This sampler can thereby give indications of where in the airways the bioaerosols would be deposited. The average measuring time was 10 min and the sampler was placed at a height of 1 m. Background bioaerosol measurements were taken using GSP and ACI samplers in common areas and residents’ rooms. ACI samples were stored in a cool box and incubated directly at 37 °C for 48 h upon return to the laboratory.

For long-term passive sampling of sedimented dust, electrostatic dust cloths (EDCs; ZEEMAN, Alphen, Holland, surface exposure area of 209 cm^2^ [19 × 11 cm]) were used (*n* = 58). The EDC has previously been used for determining endotoxin (Noss et al., 2008) and microorganisms in indoor air (Frankel, Timm, et al., 2012; Madsen et al., 2012). EDCs were placed on an open surface at each sampling visit and dust was allowed to settle for 7 days. EDCs were placed at a height ranging from 0.7 to 1.8 m in staff changing rooms, hallways, laundry area, offices, common areas, and residents’ rooms.

An outdoor reference sample was taken at every sampling visit (*n* = 10) using a GSP sampler placed at a height of 1.5 m mounted with a PC filter. The sampler was placed in an outdoor area of each nursing home, such as a courtyard, and allowed to sample for the duration of the visit (average sampling time of 385 min). Temperature and relative humidity were measured inside and outside the nursing homes during sampling using Tinytag Plus Data Loggers (Gemini Data Loggers, United Kingdom; Supplementary Table S1).

Environmental surface samples were taken from armrests, tables, door handles, light switches, bed railings, and TV remotes in both common areas and residents’ rooms (*n* = 113). Sampling was done using an eSwab transport system (Copan’s Liquid Amies Elution Swab, eSwab; Copan, Brescia, Italy). The swab was moistened in the accompanying transport medium and were then rotated axially and laterally in a zigzag motion over a surface area of 10 × 10 cm (100 cm^2^).

To study bacteria on the hands, staff members had their hands swabbed at the beginning and end of the working day using eSwabs (*n* = 158). Hand samples were collected by rotating the eSwabs axially and laterally in a zigzag motion over the entire surface of each palm, using the same swabs on both the right and left hand. After sampling using eSwabs, surface and hand swabs were transferred to their container containing the transport media (1 mL), stored cold (4 °C), and processed within 24 h of sampling.

### Extraction, quantification, and identification of microorganisms

Microorganisms from GSP filters and EDC cloths were extracted in 5- and 20-mL sterile extraction solution (MilliQ water with 0.85% NaCl and 0.05% Tween80) by orbital shaking for 15 and 60 min, respectively. Samples were plated in serial dilutions on Nutrient agar (NA; Thermo Fisher Scientific Oxioid) and incubated at 25 °C for 7 days and on SA agar at 37 °C for 48 h. Extracted samples were stored at −80 °C with 20% glycerol. To quantify and identify potential human pathogenic fungi, personal GSP samples were thawed, vortexed, and a 500-µL sample was plated on Dichloran Glycerol agar (DG18; Thermo Fisher Scientific Oxoid) at 37 °C for 7 days. Surface and hand samples were eluted by vortexing the swabs in the accompanying Amies transport medium for 2 min and plated in serial dilutions on both NA and SA agar and incubated at 25 °C for 7 days and at 37 °C for 48 h, respectively. For all samples, NA agar was used to quantify bacterial concentrations, whereas SA agar was used to both quantify and identify bacteria, specifically staphylococci.

Identification of bacteria and fungi was performed using matrix-assisted laser desorption–ionization time-of-flight (MALDI-TOF) mass spectrometry (MS) on a Microflex LT mass spectrometer (Bruker Daltonics, Bremen, Germany) using the Bruker Biotyper 3.1 software with the BDAL standard library and filamentous library 1.0 (see, e.g., Møller et al., 2022 for details). MALDI-TOF identification was performed on all bacterial isolates from SA agar plates from the following nursing homes: personal GSP samples (A–C), stationary GSP samples (A–C), stationary ACI samples (A–E), EDC samples (A–B), hand swabs (A–B), and surface swabs (A–C). MALDI-TOF identification was performed on fungal isolates from personal GSP samples from all 5 nursing homes.

### Endotoxin exposure

Extracted PC filters from a subset of the personal exposure samples (SOSU assistants [*n* = 37] and SOSU helpers [*n* = 15]) were used to determine the concentration of endotoxin in the air. Samples were centrifuged for 15 min, and the supernatant was analysed for endotoxin using a chromogenic kinetic Limulus amoebocyte lysate test (Kinetic-QCL endotoxin kit, Lonza Walkersville Inc.).

### Antifungal resistance

Identified *A. fumigatus* isolates (*n* = 40) were characterized for resistance towards 4 antifungal agents (itraconazole [Sigma–Aldrich], voriconazole [Sigma–Aldrich], amphotericin B [Sigma–Aldrich], and caspofungin acetate [Sigma–Aldrich]) using the EUCAST broth microdilution method for conidia forming moulds, version EDef 9.3.2 (Arendrup et al., 2020). Antifungal agent concentrations ranged from 0.0156 to 8 mg/L for itraconazole and from 0.0625 to 16 mg/L for the 3 remaining antifungal agents. The minimum inhibitory concentration (MIC) was determined for itraconazole, voriconazole, and amphotericin B, whereas the minimum effective concentration (MEC) was determined for caspofungin acetate. Readings of MIC and MEC values were done after 48 h. Seven *A. fumigatus* isolates collected from outdoor reference samples were also analysed for resistance, but no resistance was found.

### Statistical analyses

For a detailed description, see Supplementary Analyses S1. In short, linear mixed-effect models were used for nearly all statistical models with *nursing home* and *person ID* (when relevant) included as random effects to account for variation among nursing homes and multiple testing of the same individual, respectively. Endotoxin was analysed using a Kruskal–Wallis rank sum test, and Spearman’s rank correlations were used to analyse how endotoxin correlated to the measured microbial concentrations. Significant effects were analysed with post hoc Tukey tests with a Holm’s correction to account for multiple testing. All analyses were conducted in R v.4.2.1 (R Core Team, 2022), using packages *lme4* (Bates et al., 2015), *car* (Fox and Weisberg, 2011), *multcomp* (Hothorn et al., 2008), and *RcmdrMisc* (Fox, 2020).

## Results

### Microbial exposure

**Fig. 1.**
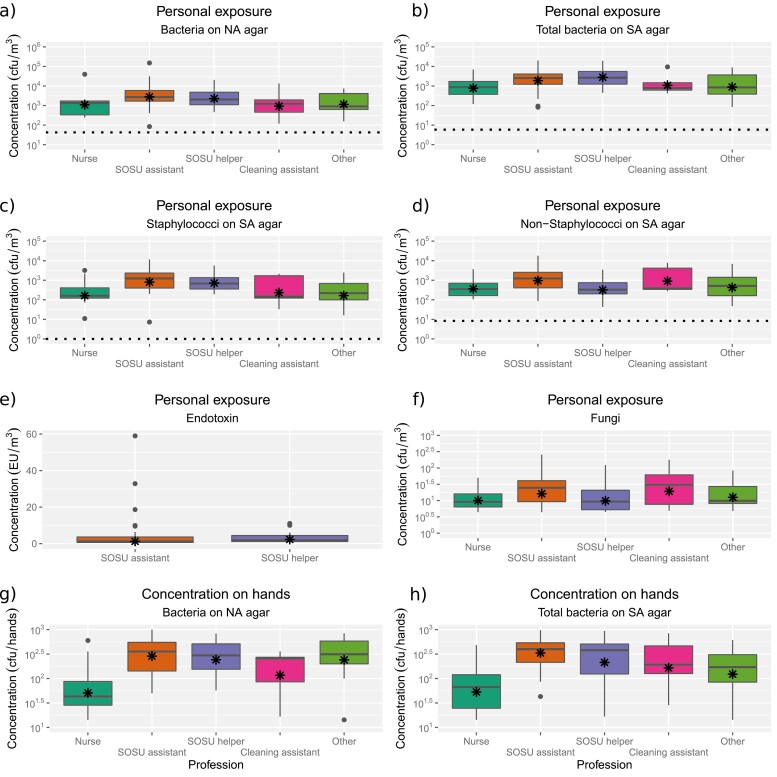
Microbial exposure among professions based on personal exposure samples (a–f) and hand swabs (g, h). Bacteria were incubated on either nutrient agar (NA) or SaSelect agar (SA), and bacteria from SA agar were further identified and grouped into either staphylococcal or non-staphylococcal bacteria. While not significant, endotoxin and fungal exposure is also illustrated (e, f). The asterisk shows the geometric mean concentration, and the dotted line shows the concentration of the outdoor references where available (for endotoxin the outdoor reference (0.3 EU/m^3^) is below the y-axis range).

GM bacterial concentrations in personal exposure samples were 2,159 cfu/m^3^ (range: 84 to 1.5 × 10^5^) for bacteria cultivated on NA agar and 1,745 cfu/m^3^ (82 to 2.0 × 10^4^) for total bacteria cultivated on SA agar. Based on MALDI-TOF identifications of bacteria on SA plates, the GM concentration of *Staphylococcus* in the personal exposure samples was 542 cfu/m^3^ (below detection [bd] limit to 1.2 × 10^4^), while the GM concentration of the remaining non-*Staphylococcus* bacteria on the SA plates was 739 cfu/m^3^ (43.3 to 1.8 × 10^4^). Bacterial exposure was typically highest for SOSU assistants and SOSU helpers (Table 1, Fig. 1).

**Table 1.** Personal exposure to airborne microorganisms and bacterial concentrations on hands grouped by profession. Presented are results from statistical models, geometric mean concentrations, ranges (in brackets), and the number of samples, *n*. For personal GSP samples, the number of samples n is shown first for general and total bacteria on NA and SA agar and next (as indicated by a forward slash) for *Staphylococcus* and non-*Staphylococcus* on SA agar. Overall differences among professions were investigated by linear mixed-effect models followed by multiple comparisons using Tukey’s test with a Holm’s correction. Bacteria were incubated on either a non-selective nutrient agar (NA) or the selective media SaSelect agar (SA). Bacteria from SA agar were further identified and grouped into either staphylococcal or non-staphylococcal bacteria. Significant *P*-values (*P* < 0.05) highlighted in bold.

	Statistical analyses		Geometric mean concentrations and ranges				
	Differences among professions	Tukey-test adjusting for multiple testing	Cleaning assistant	Nurse	Other	SOSU assistant	SOSU helper
Description	*P*-value	Comparison and *P*-value	cfu/m^3^				
GSP personal			*n* = 7/5	*n* = 8/8	*n* = 12/9	*n* = 37/29	*n* = 15/6
General bacteria (NA agar)	**0.015**	ns	1,068 (120 to 1.35 × 10^4^)	1,261 (241 to 3.98 × 10^4^)	1,296 (156 to 7,506)	30,960 (84 to 1.51 × 10^5^)	2,472 (466 to 2.04 × 10^4^)
Total bacteria (SA agar)	**0.030**	ns	1,144 (421 to 9524)	856 (120 to 6997)	1,031 (85 to 8959)	2,089 (82 to 2.01 × 10^4^)	3,042 (449 to 1.94 × 10^4^)
*Staphylococcus* (SA agar)	**0.008**	SOSU ass. vs Other *P* = **0.035** SOSU ass. vs Nurse *P* = 0.076	294 (33 to 2,147)	218 (11 to 3,207)	204 (16 to 2,484)	994 (7 to 1.15 × 10^4^)	790 (192 to 5,643)
Non-*Staphylococcus* (SA agar)	**0.015**	SOSU ass. vs Other *P* = 0.067 SOSU ass. vs Nurse *P* = 0.098	1,030 (274 to 7,738)	407 (106 to 3,693)	514 (47 to 6,941)	1,065 (86 to 1.78 × 10^4^)	375 (43 to 3,461)
Fungi	0.343	ns	24.9 (5 to 178)	11 (4 to 50)	15 (5 to 84)	19.5 (4 to 257)	11.4 (4 to 123)
**Hand swabs**			**cfu/hands**				
			***n* = 14**	***n* = 16**	***n* = 24**	***n* = 74**	***n* = 30**
General bacteria (NA agar)	**<0.001**	Nurse vs Cleaning ass. *P* = **0.002** Nurse vs. Other *P* < **0.001** Nurse vs SOSU ass. *P* < **0.001** Nurse vs SOSU help. *P* < **0.001**	342 (17 to 4,129)	44.9 (7 to 600)	523 (14 to 7,500)	950 (50 to 2.70 × 10^4^)	669 (57 to 8,829)
Total bacteria (SA agar)	**<0.001**	Nurse vs Cleaning ass. *P* = **0.048** Nurse vs Other *P* = **0.010** Nurse vs SOSU ass. *P* < **0.001** Nurse vs SOSU help. *P* = **0.004**	351 (29 to 2,743)	74 (14 to 1,229)	420 (14 to 1.67 × 10^4^)	762 (43 to 1.50 × 10^4^)	526 (17 to 1.20 × 10^4^)
*Staphylococcus* (SA agar)	—	—	139 (20 to 1,640)	55 (20 to 1,120)	212 (20 to 4,450)	406 (40 to 3,500)	186 (20 to 2,800)
Non-*Staphylococcus* (SA agar)	—	—	136 (20 to 2,100)	74 (20 to 280)	75 (10 to 1,700)	250 (20 to 2,250)	292 (60 to 2,250)

For Tukey-test are only shown comparison results where the *P*-value was below 0.1. ns = no significant differences. GSP = Gesamtstaubprobennahme sampler. A dash indicates that the measure was not analysed statistically.

Fungal GM concentrations from personal samples were 16 cfu/m^3^ air (bd to 257). No significant differences were found among professions (Table 1, Fig. 1).

An overview of concentrations of bacteria taken with stationary samplers during various work tasks can be found in Table 2. The concentration of *Staphylococcus* bacteria was elevated during bed making compared to background measurements in both the common area and residents’ rooms (Table 2). For stationary sampling with GSP samplers, no significant differences were found among different tasks.

**Table 2.** Work task and background concentrations of airborne bacteria. Presented are results from statistical models, geometric mean concentrations, ranges (in brackets), and the number of samples, *n*. For stationary GSP samples the number of samples *n* is shown first for general and total bacteria on NA and SA agar and next (as indicated by a forward slash) for *Staphylococcus* and Non-*Staphylococcus* on SA agar. Overall differences among background measures and tasks were investigated by linear mixed-effect models followed by multiple comparisons using Tukey’s test with a Holm’s correction. Bacteria were incubated on either a non-selective nutrient agar (NA) or the selective media SaSelect agar (SA). Bacteria from SA agar were further identified and grouped into either staphylococcal or non-staphylococcal bacteria. Significant *P*-values (*P* < 0.05) highlighted in bold.

	Statistical analyses		Geometric mean concentrations and ranges				
	Differences among tasks	Tukey-test adjusting for multiple testing	Background, common area	Background, resident	Bed making	Cleaning tasks	Nursing and care tasks
Description	*P*-value	Comparison and *P*-value	cfu/m^3^				
ACI stationary			*n* = 9	*n* = 8	*n* = 2	*n* = 15	*n* = 8
Total bacteria (SA agar)	0.116	ns	234 (35 to 651)	226 (53 to 969)	775 (471 to 1276)	309 (111 to 802)	277 (102 to 894)
*Staphylococcus* (SA agar)	**0.003**	Bed making vs Background, common area *P* = **0.009** Bed making vs Background, residents’ rooms *P* = **0.002** Bed making vs Cleaning tasks *P* = 0.065 Bed making vs Nursing and care tasks *P* = 0.065	52 (14 to 226)	38 (7 to 219)	470 (215 to 1028)	85 (12 to 200)	79 (14 to 326)
Non-*Staphylococcus* (SA agar)	0.994	ns	143 (18 to 545)	126 (7 to 857)	91 (39 to 215)	170 (24 to 714)	169 (57 to 799)
**GSP stationary**			***n* = 11/7**	***n* = 8/4**	***n* = 2/1**	***n* = 13/10**	***n* = 12/10**
General bacteria (NA agar)	0.832	ns	326 (22 to 2,738)	284 (48 to 2,905)	233 (143 to 381)	506 (48 to 6429)	272 (48 to 1,286)
Total bacteria (SA agar)	0.621	ns	197 (22 to 1,929)	127 (24 to 857)	700 (381 to 1286)	153 (24 to 1571)	251 (24 to 3,143)
*Staphylococcus* (SA agar)	—	—	164 (143 to 214)	571 (571 to 571)	429	267 (71 to 1,857)	254 (143 to 571)
Non-*Staphylococcus* (SA agar)	0.899	ns	637 (286 to 2,000)	912 (571 to 1,429)	571	514 (71 to 2,143)	390 (71 to 6,000)

For Tukey-test are only shown comparison results where the *P*-value was below 0.1. ns = no significant differences. ACI = Andersen Cascade Impactor (values from ACI samples are sums of all the 6 stages). GSP = Gesamtstaubprobennahme sampler. A dash indicates that the measure was not analysed statistically.

**Fig. 2.**
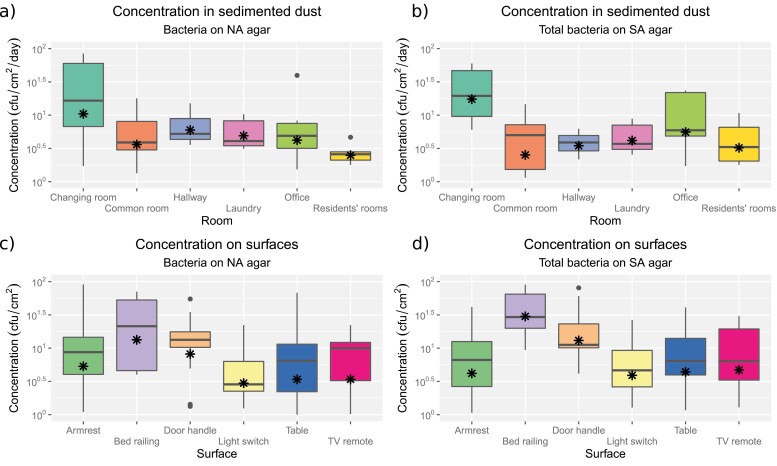
Bacterial concentrations among rooms and surfaces in sedimented dust samples (a, b) and from surface swabs (c, d). Bacteria were incubated on either nutrient agar (NA) or SaSelect agar (SA). The asterisk shows the geometric mean concentration.

Concentrations of sedimented bacteria from dust cloths showed GM concentrations of sedimented bacteria of 2 cfu/cm^2^/day (bd to 84) on NA agar and 1 cfu/cm^2^/day (bd to 60) on SA agar. For those identified, the GM of *Staphylococcus* and non-*Staphylococcus* species were 1 cfu/cm^2^/day (0.1 to 21) and 1 cfu/cm^2^/day (bd to 73), respectively. Concentrations of sedimented bacteria were highest in staff changing rooms compared to background measurements in common areas, residents’ rooms, and in the laundry area (Table 3, Fig. 2).

**Table 3.** Rooms of the nursing homes and concentrations of sedimented bacteria. Presented are results from statistical models, geometric mean concentrations, ranges (in brackets), and the number of samples, *n*. The number of samples *n* is shown first for general and total bacteria on NA and SA agar and next (as indicated by a forward slash) for *Staphylococcus* and Non-*Staphylococcus* on SA agar. Overall differences among rooms were investigated by linear mixed effect models followed by multiple comparisons using Tukey’s test with a Holm’s correction. Bacteria were incubated on either a non-selective nutrient agar (NA) or the selective media SaSelect agar (SA). Bacteria from SA agar were further identified and grouped into either staphylococcal or non-staphylococcal bacteria. Significant *P*-values (*P* < 0.05) highlighted in bold.

	Statistical analyses		Geometric mean concentrations and ranges					
	Differences among rooms	Tukey-test adjusting for multiple testing	Changing room	Common area	Hallway	Laundry	Office	Residents’ rooms
Description	*P*-value	Comparison and *P*-value	cfu/cm^2^/day					
EDC			*n* = 9/ 4	*n* = 14/ 6	*n* = 5/ 2	*n* = 10/ 4	*n* = 10/ 4	*n* = 10/ 4
General bacteria (NA agar)	**0.002**	Changing room vs Common area *P* = **0.001** Changing room vs Residents’ rooms *P* = **0.004**	7.9 (0.4 to 84.4)	1.4 (0.2 to 17.9)	2.5 (0.6 to 15.1)	2.2 (0.1 to 10.3)	2.2 (0.3 to 39.6)	1.2 (0.1 to 4.7)
Total bacteria (SA agar)	**0.008**	Changing room vs Common room *P* = **0.006** Changing room vs Residents’ rooms *P* = **0.001** Changing room vs Laundry *P* = **0.010**	5.1 (0.2 to 59.8)	1.3 (0.1 to 14.6)	1.3 (0.1 to 6.3)	0.7 (0.1 to 8.9)	1.3 (0.05 to 23.4)	0.7 (0.05 to 10.8)
*Staphylococcus* (SA agar)	—	—	16.7 (13.2 to 21.1)	0.3 (0.2 to 0.4)	1.1 (1.1 to 1.1)	0.6 (0.1 to 3.1)	0.2 (0.2 to 0.4)	2.5 (1.2 to 9.4)
Non-*Staphylococcus* (SA agar)	—	—	6.6 (0.2 to 72.7)	0.6 (0.2 to 1.7)	4.5 (2.8 to 7.3)	0.2 (0.05 to 1.0)	2.1 (0.5 to 9.2)	0.5 (0.05 to 4.8)

A dash indicates that the measure was not analysed statistically.

GM bacterial concentrations on surfaces were 5 cfu/cm^2^ (0.1 to 373) for bacteria on NA, 4 cfu/cm^2^ (bd to 750) for both bacteria on SA and staphylococci, and 1 cfu/cm^2^ (bd to 100) for non-staphylococci. Bacterial concentrations were highest on bed railings and lowest on light switches, and were overall highest in common areas compared with residents’ rooms (Table 4, Fig. 2).

**Table 4.** Surface concentrations of bacteria in common areas and residents’ rooms. Presented are results from statistical models, geometric mean concentrations, ranges (in brackets), and the number of samples, *n*. The number of samples *n* is shown first for general and total bacteria on NA and SA agar and next (as indicated by a forward slash) for *Staphylococcus* and Non-*Staphylococcus* on SA agar. Overall differences among areas (common areas and residents’ rooms) and surface types were investigated by linear mixed effect models followed by multiple comparisons using Tukey’s test with a Holm’s correction for surface types. Bacteria were incubated on either a non-selective nutrient agar (NA) or the selective media SaSelect agar (SA). Bacteria from SA agar were further identified and grouped into either staphylococcal or non-staphylococcal bacteria. Significant *P*-values (*P* < 0.05) highlighted in bold.

		Statistical analyses		Geometric mean concentrations and ranges							
				Area		Surface type					
	Differences among areas	Differences among surface types	Tukey-test adjusting for multiple testing	Common area	Residents’ rooms	Armrest	Bed railing	Door handle	Light switch	Table	TV remote
Description	*P*-value	*P*-value	Comparison and *P*-value			cfu/cm^2^/day					
Surface swabs				*n* = 52/30	*n* = 61/37	*n* = 20/12	*n* = 10/6	*n* = 23/13	*n* = 20/12	*n* = 20/12	*n* = 20/12
General bacteria (NA agar)	**<0.001**	**<0.001**	Bed railing vs Armrest *P* = **0.045** Bed railing vs Door handle *P* = **0.042** Bed railing vs Table *P* = **0.006** Bed railing vs TV remote *P* = **0.006** Bed railing vs Light switch *P* < **0.001** Light switch vs Door handle *P* = **0.025** Light switch vs Armrest vs *P* = **0.032**	8.4 (0.3 to 373)	2.9 (0.1 to 111)	6.3 (0.5 to 91)	15.4 (0.8 to 111)	6.4 (0.2 to 373)	1.7 (0.1 to 22)	4.3 (0.3 to 104)	4.2 (0.2 to 22)
Total bacteria (SA agar)	**<0.001**	**<0.001**	Bed railing vs Armrest *P* = **0.026** Bed railing vs Door handle *P* = **0.024** Bed railing vs Table *P* < **0.001** Bed railing vs TV remote *P* = **0.004** Light switch vs Bed railing *P* < **0.001** Light switch vs Armrest *P* = 0.080 Light switch vs Door handle *P* = **0.066**	6.5 (0.02 to 750)	2.9 (0.06 to 227)	6.1 (0.7 to 140)	27.1 (0.2 to 227)	6.3 (0.07 to 750)	1.5 (0.04 to 27)	2.1 (0.02 to 41)	4.0 (0.3 to 30)
*Staphylococcus* (SA agar)	**0.023**	0.082	ns	2.3 (0.02 to 750)	0.9 (0.02 to 71)	1.5 (0.02 to 20)	4.4 (0.02 to 71)	3.3 (0.2 to 750)	0.5 (0.02 to 5)	0.9 (0.02 to 18)	1.6 (0.02 to 30)
Non-*Staphylococcus* (SA agar)	0.157	0.817	ns	1.9 (0.02 to 37)	0.9 (0.02 to 100)	2.4 (0.2 to 100)	1.6 (0.2 to 22)	0.8 (0.02 to 55)	1.3 (0.05 to 22)	1.0 (0.02 to 23)	1.4 (0.02 to 41)

ns = no significant differences.

Bacterial concentrations on individual staff members’ hands were significantly higher at the start of the working day compared with the end of the working day (NA agar: *P* = 0.011, SA agar, *P* = 0.033). GM for bacteria on NA agar at the start of the day was 693 cfu/hands (bd to 2.7 × 10^4^) and at the end of the day 427 cfu/hands (14 to 8,800), GM for total bacteria on SA agar at the start of the day was 579 cfu/hands (14 to 1.5 × 10^4^) and at the end of the day 396 cfu/hands (14 to 1.7 × 10^4^). Nurses had the lowest bacterial concentrations on hands and SOSU assistants the highest (Table 1, Fig. 1).

MALDI-TOF identification was attempted on bacterial isolates from SA agar from the stationary ACI air samples (*n* = 4,662), stationary GSP air samples (*n* = 262), outdoor reference air samples (*n* = 42), personal GSP air samples (*n* = 3337), surfaces samples (*n* = 2973), EDC samples (*n* = 659), and hand swabs (*n* = 2,019). Of these isolates, a successful identification to genus level was obtained for 94.9%, 90.5%, 76.2%, 94.1%, 95.5%, 95.0%, and 96.0%, respectively. Staphylococci amounted to 29.6%, of the isolates from the stationary ACI air samples, 21.4% of the stationary GSP air samples, 0% of the outdoor reference air samples, 43.1% of the personal GSP air samples, 45.4% of the surfaces samples, 30.4% of the EDC samples, and 63.5% of the hand swabs. From the different sample types, we identified a total of 71 bacterial species, with most species from the genera *Staphylococcus*, *Corynebacterium*, and *Bacillus* (Supplementary Table S3).

Identifications of fungi to species level were successful for 148 fungal isolates (of 171 isolates, Supplementary Table S3). Thirteen species were identified, and the most commonly found species were *Aspergillus niger* (47 isolates), *A. fumigatus* (40 isolates), *Penicillium chrysogenum* (16 isolates), and *A. ustus* (15 isolates). Aside from these, we identified (in descending order) *P. citrinum*, *A. candidus*, *A. nidulans*, *Candida orthopsilosis*, *Paecilomyces variotii*, *C. parapsilosis*, *Microsporum equinum*, *P. funiculosum*, and *P. glabrum*.

### Endotoxin exposure

The GM exposure to endotoxin was 1.5 EU/m^3^, ranging from 0.02 to 59.0 EU/m^3^. Endotoxin exposure did not differ between professions (χ^2^ = 1.97, df = 1, *P* = 0.160; Fig. 1). Endotoxin exposures were significantly positively correlated with the total bacterial exposure on SA agar (*r* = 0.49, *P* = 0.023).

### Antifungal resistance

Of the 40 *A. fumigatus* isolates tested, resistance was found in 2 isolates from SOSU assistants. These were found from 2 different nursing homes. One isolate was resistant towards both itraconazole (MIC > 8 mg/mL) and voriconazole (MIC of 2 mg/mL), and one isolate was resistant towards amphotericin B (MIC of 2 mg/mL). For the remaining isolates, average (mean) MIC values were for itraconazole 0.345 mg/mL, for voriconazole 0.455 mg/mL, and for amphotericin B 0.503 mg/mL. For caspofungin acetate, the average MEC value was 0.419 mg/mL. See Supplementary Table S4 for a full list of MIC values.

## Discussion

### Exposure to bacteria and factors influencing exposure

Across all sample types, a total of 71 bacterial species were identified from SA agar (a selective agar medium mainly targeting *Staphylococcus* species), of which 17 were from the genus *Staphylococcus*, 15 were from the genus *Corynebacterium*, and 10 were from the genus *Bacillus*. Both staphylococci and corynebacteria are known to be associated with the human skin microbiome (Kloos and Musselwhite, 1975; Dekio et al., 2005; Gao et al., 2007) and are typical of indoor environments (Rintala et al., 2008; Täubel et al., 2009; Hospodsky et al., 2012; Madsen et al., 2018, 2023). The presence of a large portion of these are likely due to a large number of staff and residents working and living closely together. For example, in a study comparing how human occupancy in a university classroom can act as a source of indoor bacteria, Hospodsky et al. (2012) found that staphylococci and corynebacteria were some of the main genera in indoor air and floor dust. The ratio of staphylococci to other bacteria differed among sample types, though this is to a large degree based on the agar medium used, which selects for staphylococci and inhibits most other bacterial genera. Nonetheless, the proportion of staphylococcal species was largest in samples from hands (64%), which matches the fact that most staphylococci are skin related (Dekio et al., 2005; Gao et al., 2007). Following hand swabs, most staphylococci were found in personal samplers (43%) and surface samples (45%), again matching our expectations.

Bacterial exposure levels for staff at nursing homes varied among professions from (geometric mean, GM, concentrations) around 1,000 cfu/m^3^ for cleaning assistants, nurses, and other staff, and around GM 2,500 to 3,000 cfu/m^3^ for SOSU helpers and assistants. Few studies use personal samplers, such as the GSP sampler, for determining airborne exposure to microorganisms in indoor occupational environments. However, one study using GSP samplers conducted in Danish homes showed that the GM personal bacterial exposure of each season varied between 300 cfu/m^3^ in summer to 1.6 × 10^4^ cfu/m^3^ in autumn (Madsen et al., 2018). Frankel, Bekö, et al. (2012) also found significant seasonal variation in several measures of indoor microbial exposure, including bacteria, in Danish homes. Here the authors found the highest bacterial concentrations in spring (median 2,165 cfu/m^3^) and lowest in summer (median 240 cfu/m^3^), though this was quantified using stationary GSP samplers. While the GM concentrations among professions varied from 1,000 to 3,000 cfu/m^3^, exposures for some staff members reached levels up to 3.9 × 10^4^ cfu/m^3^ (for nurses) and 1.5 × 10^5^ cfu/m^3^ (for SOSU assistants). Microbial concentrations in indoor environments have also been examined using EDCs, which measure the sedimented dust levels. In the study on Danish homes, Madsen et al. (2018) found GM bacterial concentrations of 1,900 cfu/m^2^/day. In the present study, we found GM concentrations of bacteria of around 10,000 to 20,000 cfu/m^2^/day—a factor 10 higher. This may be a product of several factors, such as the higher occupancy in nursing homes, more people present throughout the day at the nursing homes compared to regular homes where people are away at work, the tasks performed in nursing home which may generate or resuspend bacteria, or a lack of ventilation. The larger number of people can act as a source of bacteria, e.g., from the skin, and more bacteria can be resuspended from, e.g., the floor during activities and then settle on surfaces.

Microbial exposure differed significantly among professions, with the highest exposures seen for SOSU assistants and SOSU helpers and the lowest for nurses and persons in “other” jobs, such as trainees and dieticians. The differences among exposures are likely due to the differences in the typical tasks performed (see Supplementary Table S2 for an overview of job descriptions), which for SOSU assistants and helpers mainly include personal care and nursing tasks and which for nurses at nursing homes in Denmark includes more administrative tasks. SOSU assistants’ and helpers’ tasks can also include bed making, which matches results taken with stationary samplers which showed elevated levels of *Staphylococcus* bacteria during this task. However, it is worth noting that only a few samples were collected during bed making and that stationary short-term sampling may either underestimate or overestimate the actual exposure depending on the distance to the source. Nonetheless, during bed making, bacteria from persons’ skin, such as staphylococci, and other dust particles can get resuspended from the fabric and dust and into the air (Ferro et al., 2004; Qian and Ferro, 2008; Qian et al., 2014; Lewis et al., 2018). Similar to this, we found elevated concentrations of sedimented microorganisms in staff changing rooms compared to common areas and residents’ rooms. These elevated concentrations may be due to the resuspension of bacteria from clothes when staff members are changing. Matching that, sedimented dust concentrations were lowest in the residents’ rooms, which most of the time only contained one occupant. Bacterial concentrations also differed among different surfaces, as measured using swabs. While all surfaces sampled are considered high-level contact points, the highest bacterial concentrations were found on bed railings in residents’ rooms but was generally highest in common areas. Bacterial concentrations on surfaces measured using NA agar showed concentrations up to 373 cfu/cm^2^. This is higher than measured on desks using the same methodology, where the authors found concentrations ranging from 1.4 to 5.0 cfu/cm^2^. Evidence shows that contaminated surfaces can play a key role in the transmission of pathogens. For example, hand contamination in health care personnel happens just as frequently through direct contact with an infected or colonized individual as when the personnel are in contact with contaminated surfaces (Stiefel et al., 2011).

Higher bacterial concentrations on hands were found at the start of the working day compared to at the end of the working day. This is likely due to increased hand hygiene at work, potentially due to the increased focus on hand hygiene in the working place in a health care environment as well as increased focus on hand hygiene in more recent times. In contrast to this study, in a study from 1999, the authors found an increase in bacterial concentrations in health care workers as the day progressed (Pittet et al., 1999). The authors found between 0 and 300 cfu/hand (5 fingertips imprinted on commercial contact plates), whereas we took swabs from the palms and found an average concentration at the end of the working day of 200 cfu/hand. Differences in bacterial concentrations in the present study were also found among professions, indicating different levels of hand hygiene or differences in the level of personal contact with residents. For example, the lowest bacterial concentrations were found on nurses’ hands who have more administrative tasks, whereas the highest was found on SOSU assistants who have more direct contact with residents during care and nursing tasks. Matching these findings, in a study by Gaspard et al. (2010), the authors found that social and healthcare assistants had a higher bacterial concentration on their forearms from personal care tasks with patients than for example nurses and cleaners.

### Exposure to endotoxin

Endotoxin exposure levels were determined in SOSU assistants and SOSU helpers, but did not differ among the 2 professions. This is likely because they have similar job functions and because the endotoxin exposure in personal air samples is very task dependent and can differ from day to day. For example, the highest exposure was found in one SOSU assistant who, as one of the tasks that day, cleaned a resident’s bird cage. Aside from the high exposure during bird cage cleaning, levels of endotoxin were comparable to other indoor studies and suggests that normal everyday tasks do not lead to elevated exposure. In a study from Korea, the authors found that endotoxin levels in the lobby of a hospital varied between the limit of detection and 7 EU/m^3^. They found that endotoxin levels correlated to the total suspended particulate matter, carbon dioxide, the number of visitors per day, and the time spent on cleaning (Lee et al., 2014).

### Fungal exposure

The concentration of potential fungal pathogens, i.e. those fungi able to grow at 37 °C, was also determined and was found to be fairly low (GM = 16 cfu/m^3^) and comparable to outdoor concentrations (GM = 13 cfu/m^3^). No differences among professions were found. The concentrations determined are markedly lower than in other studies, though most studies rarely examine the indoor fungal exposure for fungi able to grow at 37 °C. For example, in a study of nursing homes in Turkey, Yilmaz et al. (2017) incubated samples at room temperature and found a vastly different concentration and composition of fungi. Nonetheless, some of the species found in the study in Turkey also occur in the present study, such as *Aspergillus niger*, *A. fumigatus*, and *Penicillium chrysogenum*, which were the 3 most common fungal species in this study. These species are often found in indoor air. The species found also match that of other studies with samples incubated at 25 °C (Knudsen et al., 2017). Interestingly, Lu et al. (2020) has shown that *A. fumigatus* and *A. niger* have high cytotoxic potentials, indicating that inhalation of these fungi may cause health problems in staff members.

### Antifungal resistance

*Aspergillus fumigatus* is known to cause serious infections, especially in occupational settings (Latgé, 1999; Poole and Wong, 2013). Because of the problematic infections it can cause and subsequent treatment, it is also a species of fungi often found to be resistant to antimicrobial agents. We tested 40 *A. fumigatus* isolates for resistance towards 3 different classes of antimicrobials and found a low incidence of resistance, with 2 isolates showing resistance. One isolate was multiresistant to both itraconazole and voriconazole, a class of antimicrobials that are typically used as a first line of defence. The other isolate showed resistance towards amphotericin B, which is an older antimicrobial known for its many side effects. This drug is often used if fungal infections show resistance towards azole treatment. Studies indicate that development of resistance due to treatment is uncommon (Moosa et al., 2002; Dannaoui et al., 2004); however, Ashu et al. (2018) found widespread amphotericin B resistance (96%) in *A. fumigatus* isolates from Hamilton, Canada.

## Conclusions

We studied the occupational microbial exposure in 5 nursing homes in the Capital Region of Denmark, where we focused on examining the exposure via several routes: personal airborne exposure, contact with surfaces, hand hygiene, and dispersal of microorganisms during work tasks sampled by stationary samplers. Exposure to endotoxin and potentially harmful and antimicrobial-resistant fungi was low, and endotoxin exposure were comparable to typical indoor levels, while fungal exposure was on level with outdoor reference values. Bacterial and staphylococcal exposure were for many samples comparable to levels in homes, but could reach elevated levels of up to 1.5 × 10^5^ cfu/m^3^. Bacterial exposures were higher for professions with more direct contact with the residents, i.e., social and healthcare (SOSU) assistants and helpers, whose main tasks include personal care and nursing task, compared to nurses, who in Danish nursing homes have more administrative tasks. Other areas of elevated bacterial concentrations, which may be areas in which to increase hygiene, is surfaces in common areas, bed railings in residents’ rooms, and sedimented dust in staff changing rooms. Bacterial concentrations on SOSU assistants’ and SOSU helpers’ hands were high compared to other professions, and increased focus on hand hygiene for these employees is likely of value in the future.

## Supplementary Material

wxad032_suppl_Supplementary_Material

## Data Availability

Data underlying this article will be shared on reasonable request to the corresponding author.
