# Supplementary material for: Work in nursing homes and occupational exposure to endotoxin and bacterial and fungal species
Source: Ann Work Expo Health. 2023 Jun 10;67(7):831–46. doi: 10.1093/annweh/wxad032 (PMC10410494; doi:10.1093/annweh/wxad032)
Supplement: wxad032_suppl_Supplementary_Material [file wxad032_suppl_supplementary_material.docx]

**Supplementary Information**

**Work in nursing homes and occupational exposure to endotoxin and bacterial and fungal species**

**Pil Uthaug Rasmussen, Katrine Uhrbrand, Margit W. Frederiksen, Anne Mette Madsen**

**Analyses S1**

Bacterial concentrations were calculated as geometric mean (GM) concentration per m^3^ of air (cfu/m^3^) for GSP and ACI air samples, as GM concentration per cm^2^ per day (cfu/cm^2^/day) for EDC samples, as GM concentration per cm^2^ (cfu/cm^2^) for surface samples, and as GM concentration per total surface of hands (cfu/hands). For bacterial concentrations below the limit of detection (LOD) a concentration corresponding to 0.5 x LOD was allocated. Limits of detection was on average: personal GSP = 8.2 cfu/m^3^ bacteria, 10.8 cfu/m^3^ fungi, endotoxin = 0.02 EU/m^3^, GSP during tasks = 47.6 cfu/m^3^, ACI during tasks = 3.5 cfu/m^3^, EDC = 0.1 cfu/cm^2^/day, surface = 0.02 cfu/cm^2^, hands = 14.3 cfu/hands, and outdoor references = 1.2 cfu/m^3^.

Personal GSP samples: In order to investigate the impact of profession on microbial exposures, we ran linear mixed effect models with *profession* as a fixed effect. To account for multiple sampling of the same person, we included *person ID* as random effects. Nursing homes were visited twice with two weeks between visits and the sampling ran from autumn to summer. Therefore, the terms *nursing home* and *season* were to a large part confounded. In the models, we included *nursing home* as a random effect to account for variation between visits, as this gave a better model (based on AIC scores) than *season*.

Personal hand swabs: In order to investigate the impact of profession and time of day (start or end of the working day), we ran linear mixed effect models with *profession* and *time* as fixed effects. We included *person ID* and *nursing home* as random effects.

Stationary GSP and ACI samples: In order to investigate the impact of work task on microbial concentrations, we ran linear mixed effect models with *work task* as a fixed effect. The factor *nursing home* was included as a random effect.

Stationary EDC samples: In order to investigate the impact of room on microbial concentrations, we ran linear mixed effect models with *room* as a fixed effect. The factor *nursing home* was included as a random effect.

Surface samples: In order to investigate the impact of area (common area or residents’ rooms) and surface type on microbial concentrations, we ran linear mixed effect models with *area* and *surface* as fixed effects. The factor *nursing home* was included as a random effect.

Endotoxin: As endotoxin concentrations did not meet model assumptions of linear mixed effect models, we instead used Kruskal-Wallis rank sum tests to examine the effect of *profession*. The correlation between endotoxin and microbial concentrations (using data from personal exposure samples in nursing home A-C) was explored using Spearman’s rank correlations using a Holm’s correction to account for multiple comparisons.

All microbial concentrations were log-transformed and model assumptions were examined. For models on *Staphylococcus* and non-*Staphylococcus* concentrations from SA agar plates, we used the subset of data in which these groups had been identified (if this included three or more nursing homes). Significant effects in all models with more than two levels were explored by posthoc Tukey tests using a Holms correction for multiple comparisons.

**Table S1** The number of samples per nursing home, as well as the temperature and relative humidity measured inside and outside the nursing homes (A-E). Measurements were taken during 14 days for indoor measurements and from morning until afternoon on the sampling day for outdoor measurements. Shown are means ± sd.

| Nursing  home | Number of participants  (visit 1 + visit 2) | Sampling times |  | Indoor | |  | Outdoor | |
| --- | --- | --- | --- | --- | --- | --- | --- | --- |
|  |  |  |  | Temperature | Relative humidity |  | Temperature | Relative humidity |
|  |  |  |  | °C | % |  | °C | % |
| A | 11 + 11 | Autumn |  | 23.4 ± 1.4 | 49.1 ± 9.8 |  | 14.8 ± 1.2 | 85.9 ± 6.1 |
| B | 9 + 7 | Autumn-Winter |  | 20.8 ± 0.8 | 39.1 ± 5.7 |  | 2.0 ± 1.1 | 98.0 ± 2.1 |
| C | 9 + 10 | Winter |  | 21.0 ± 0.5 | 44.8 ± 3.9 |  | 0.3 ± 2.1 | 78.1 ± 14.8 |
| D | 7 + 4 | Spring |  | 23.8 ± 0.8 | 40.2 ± 5.6 |  | 14.7 ± 5.1 | 53.1 ± 19 |
| E | 6 + 5 | Summer |  | 24.7 ± 1.2 | 45.0 ± 5.7 |  | - | - |
| Total | 79 |  |  |  |  |  |  |  |

Information regarding the outdoor temperature and relative humidity during sampling at nursing home E were not available due to a technical error.

**Table S2** Overview of typical work tasks by profession for social and health care (SOSU) assistants, SOSU helpers, nurses, cleaning assistants, and others.

| Profession | Number of samples  from participants in study | Total number in Denmark (in 2017)* | Length of education | Typical daily tasks in nursing homes |
| --- | --- | --- | --- | --- |
| SOSU assistant | 37 | 53.300 | 3 years and 10 months | Provide nursing care and personal care, incl. administering medicine  Potential smaller cleaning tasks incl. bed making  Support and guide residents who are ill or undergoing treatment  Talk to and guide residents in difficult life situations  Help with rehabilitation after, for example, an injury or illness  Collaboration across professional groups |
| SOSU helper | 15 | 60.800 | 2 year and 2 months | Help residents with visits to the toilet, bathing and dressing  Potential smaller cleaning tasks incl. bed making  Help with rehabilitation  Create a good relationship with the citizen  Use digital aids and welfare technology  Notice changes in the citizen's health and well-being |
| Nurse | 8 | 71.200 | 3 years and 6 months | Administrative tasks, such as:  Provide qualified medical knowledge  Oversee resident action plans |
| Cleaning assistant | 7 | n/a | 1 year and 6 months to 2 years | Various cleaning task, including vacuuming |
| Other | 12 | n/a |  |  |

* *Other* includes SOSU assistant trainees, substitute/temporary workers, dieticians, and ‘activity-staff’ i.e. persons doing activities with the residents.

* Reference: Sundhedsdatastyrelsen. 2020. SOSU- og sygeplejerskeuddannede i Danmark 2000-2017 - Arbejdsstyrke, beskæftigelse og tilbagetrækning. [*SOSU and nursing graduates in Denmark 2000-2017 - Workforce, employment and retirement*]

**Table S3** Heatmap of bacterial and fungal species found in personal Gesamtstaubprobennahme (GSP) samplers, stationary GSP samplers, stationary Andersen Cascade Impactors (ACI), electrostatic dust collectors (EDC), surface swabs, and hand swabs. Values refer to geometric mean concentrations of positive samples.

|  | **GSP** | **GSP** | **ACI** | **EDC** | **Surface** | **Hands** |
| --- | --- | --- | --- | --- | --- | --- |
|  | **Person** | **Stationary** | **Stationary** |  |  |  |
|  | **cfu/m^3^** | **cfu/m^3^** | **cfu/m^3^** | **cfu/cm^2^/day** | **cfu/cm^2^** | **cfu/hands** |
| **Bacteria** |  |  |  |  |  |  |
| *Aerococcus* sp. | 89.3 |  | 3.47 |  | 2.83 | 150 |
| *Aerococcus viridans* | 92.92 |  | 4.12 | 0.14 | 0.46 |  |
| *Aeromonas* sp. |  |  |  | 0.19 |  |  |
| *Arthrobacter creatinolyticus* | 51.36 | 285.71 |  |  |  |  |
| *Arthrobacter cumminsii* |  | 428.57 |  | 0.19 | 1.73 | 20 |
| *Arthrobacter gandavensis* |  |  | 3.53 |  |  |  |
| *Arthrobacter* sp. | 25.98 | 428.57 | 4.61 | 0.38 | 2.06 | 40 |
| *Bacillus altitudinis* |  |  | 3.53 |  | 0.09 |  |
| *Bacillus cereus* | 124.46 |  | 3.73 |  | 0.25 | 20 |
| *Bacillus flexus* | 64.03 | 5.83 | 7.33 |  | 0.48 | 20 |
| *Bacillus gibsonii* | 9.46 |  |  |  |  |  |
| *Bacillus infantis* | 8.03 |  | 3.53 |  |  |  |
| *Bacillus licheniformis* |  |  | 3.41 |  | 0.07 |  |
| *Bacillus marisflavi* |  |  | 3.53 |  |  |  |
| *Bacillus megaterium* |  |  | 3.53 |  |  |  |
| *Bacillus pumilus* | 11.81 |  | 2.94 |  | 0.06 |  |
| *Bacillus* sp. | 45.9 | 12.4 | 4.53 | 0.19 | 0.26 |  |
| *Bacillus subtilis* |  |  | 3.84 |  |  |  |
| *Brevibacterium casei* | 89.36 |  | 3.44 |  | 0.45 | 72.48 |
| *Brevibacterium celere* |  | 142.86 | 4.56 |  |  |  |
| *Brevibacterium ravenspurgense* | 117.52 | 714.29 | 4.62 | 0.57 | 2.38 |  |
| *Brevibacterium* sp. | 177.39 |  | 2.94 | 0.19 | 0.52 | 60.28 |
| *Cellulosimicrobium cellulans* | 82.82 |  |  |  |  | 20 |
| *Corynebacterium afermentans* | 66.17 |  | 4.16 | 0.38 |  | 31.62 |
| *Corynebacterium ammoniagenes* |  |  | 2.94 |  |  |  |
| *Corynebacterium amycolatum* | 60.23 | 142.86 | 4.44 | 0.38 | 0.41 | 20 |
| *Corynebacterium aurimucosum* | 55.68 | 1069.05 | 7.84 | 0.58 | 1.65 | 53.13 |
| *Corynebacterium casei* |  |  |  |  |  | 20 |
| *Corynebacterium confusum* | 9.52 |  | 3.53 |  |  |  |
| *Corynebacterium coyleae* | 61.33 | 857.14 | 7.16 | 0.19 | 0.3 | 30.67 |
| *Corynebacterium freneyi* |  |  | 4.19 |  |  |  |
| *Corynebacterium imitans* | 105.06 |  | 4.77 | 0.19 | 0.24 |  |
| *Corynebacterium minutissimum* | 20.14 |  | 4.76 |  |  | 50 |
| *Corynebacterium mucifaciens* | 7.22 |  | 3.53 |  |  |  |
| *Corynebacterium riegelii* | 169.56 |  | 3.72 | 0.1 | 0.44 |  |
| *Corynebacterium simulans* |  |  |  |  | 0.2 | 50 |
| *Corynebacterium* sp. | 99.55 | 142.86 | 4.52 | 0.31 | 0.66 | 42.24 |
| *Corynebacterium stationis* | 15.36 |  | 3.47 | 0.19 |  |  |
| *Corynebacterium striatum* | 30.8 |  | 5.36 | 0.19 | 0.86 |  |
| *Dermabacter hominis* |  |  | 3.53 |  | 0.26 |  |
| *Dermabacter* sp. |  |  | 3.53 | 0.19 | 0.47 |  |
| *Dolosigranulum pigrum* |  |  |  |  | 0.1 |  |
| *Enterococcus faecalis* |  |  | 3.93 |  | 3.08 |  |
| *Exiguobacterium aurantiacum* |  |  | 3.23 |  |  |  |
| *Exiguobacterium* sp. |  |  | 6.45 |  |  |  |
| *Facklamia* sp. |  |  |  |  | 0.06 |  |
| *Jeotgalicoccus halotolerans* |  |  | 3.4 |  |  |  |
| *Jeotgalicoccus* sp. |  |  | 5.35 |  |  |  |
| *Kocuria kristinae* | 71.61 |  | 3.53 |  | 0.54 | 158.74 |
| *Kocuria palustris* | 8.03 | 3.41 | 3.53 |  |  |  |
| *Kocuria rhizophila* |  |  | 3.53 |  |  |  |
| *Kocuria* sp. | 26.99 |  | 3.53 | 0.77 | 9.8 | 31.62 |
| *Kytococcus schroeteri* |  |  | 2.94 |  |  |  |
| *Kytococcus sedentarius* |  |  |  |  |  | 250 |
| *Kytococcus* sp. |  |  | 2.94 |  |  |  |
| *Lactobacillus coleohominis* |  |  |  | 0.19 |  |  |
| *Lactobacillus* sp. |  |  |  | 0.19 |  |  |
| *Leuconostoc* sp. | 9.96 |  |  |  |  |  |
| *Lysinibacillus fusiformis* |  |  | 3.53 |  |  |  |
| *Macrococcus caseolyticus* |  |  | 3.23 |  |  |  |
| *Macrococcus* sp. |  |  | 2.94 |  |  |  |
| *Microbacterium arborescens* |  |  | 4.82 |  |  | 173.21 |
| *Microbacterium* sp. |  |  |  |  |  | 144.91 |
| *Micrococcus luteus* | 526.62 | 287.43 | 25.83 | 1.24 | 0.88 | 132.25 |
| *Micrococcus lylae* | 18.89 |  | 4.05 | 0.57 | 1.34 | 20 |
| *Micrococcus* sp. | 66.48 | 81.29 | 5.58 | 0.19 | 0.24 | 40.59 |
| *Oceanobacillus profundus* |  |  | 3.53 |  |  |  |
| *Paenibacillus* sp. |  |  | 2.94 |  |  |  |
| *Pseudoclavibacter* sp. |  |  | 2.94 |  |  |  |
| *Pseudomonas* sp. |  |  | 3.53 |  |  |  |
| *Rothia endophytica* | 23.23 |  |  |  |  | 20 |
| *Rothia nasimurium* |  |  | 2.94 |  |  |  |
| *Rothia* sp. | 30.97 |  |  |  |  | 20 |
| *Rothia terrae* | 165.15 |  |  |  | 0.24 | 100 |
| *Staphylococcus aureus* | 43.6 | 671.81 | 12.75 | 0.38 | 0.99 | 40.81 |
| *Staphylococcus auricularis* |  |  | 7.41 | 0.1 | 0.06 | 31.62 |
| *Staphylococcus capitis* | 125.71 | 303.05 | 5.16 | 0.33 | 0.39 | 76.85 |
| *Staphylococcus caprae* | 16.81 |  | 6.89 | 0.1 | 0.24 | 20 |
| *Staphylococcus cohnii* | 31.92 |  | 3.53 | 0.19 | 0.2 |  |
| *Staphylococcus epidermidis* | 128.7 | 89.99 | 6.6 | 0.69 | 0.9 | 156.66 |
| *Staphylococcus equorum* | 38.72 |  | 2.94 |  | 0.26 | 150 |
| *Staphylococcus haemolyticus* | 91.43 | 202.03 | 6.42 | 0.47 | 0.42 | 44.09 |
| *Staphylococcus hominis* | 105.06 | 101.02 | 9.19 | 0.72 | 0.56 | 70.74 |
| *Staphylococcus lugdunensis* | 9.46 |  | 3.87 | 0.1 | 0.2 | 40 |
| *Staphylococcus pasteuri* |  |  | 5.59 |  | 0.82 | 75.29 |
| *Staphylococcus pettenkoferi* | 168.99 | 285.71 | 6.2 | 0.76 | 0.85 | 37.87 |
| *Staphylococcus saprophyticus* | 32.55 |  | 5.26 | 0.35 |  | 64.51 |
| *Staphylococcus sciuri* |  |  |  |  |  | 54.77 |
| *Staphylococcus simulans* | 65.74 |  | 4.26 | 0.38 | 0.1 | 27.14 |
| *Staphylococcus* sp. | 137.4 | 179.99 | 6.26 | 0.52 | 0.44 | 58.1 |
| *Staphylococcus warneri* | 151.57 |  | 4.09 | 0.1 | 0.34 | 55.52 |
| *Staphylococcus xylosus* |  |  |  |  |  | 60 |
| *Steptomyces* sp. | 11.07 |  |  |  |  |  |
| *Turicella otitidis* |  |  |  |  |  | 53.13 |
| *Turicella* sp. | 11.81 |  |  |  |  | 27.14 |
|  |  |  |  |  |  |  |
| **Fungi** |  |  |  |  |  |  |
| *Aspergillus candidus* | 13.19 |  |  |  |  |  |
| *Aspergillus fumigatus* | 18.68 |  |  |  |  |  |
| *Aspergillus nidulans* | 9.96 |  |  |  |  |  |
| *Aspergillus niger* | 23.28 |  |  |  |  |  |
| *Aspergillus ustus* | 25.14 |  |  |  |  |  |
| *Candida orthopsilosis* | 31.65 |  |  |  |  |  |
| *Candida parapsilosis* | 18.99 |  |  |  |  |  |
| *Microsporum equinum* | 17.32 |  |  |  |  |  |
| *Paecilomyces variotii* | 9.79 |  |  |  |  |  |
| *Penicillium chrysogenum* | 19.8 |  |  |  |  |  |
| *Penicillium citrinum* | 12.51 |  |  |  |  |  |
| *Penicillium funiculosum* | 11.95 |  |  |  |  |  |

**Table S4** *Aspergillus fumigatus* isolates tested for antifungal resistance using the EUCAST broth microdilution method. The minimum inhibitory concentration (MIC) was determined for itraconazole, voriconazole, and amphotericin B, whereas the minimum effective concentration (MEC) was determined for caspofungin acetate. Whether isolates were resistant (R, highlighted in red) or susceptible (S) was determined based on EUCAST breakpoint values. Note that no breakpoints are available for Caspofungin.

|  |  | Itraconazole | | Voriconazole | | Amphotericin B | | Caspofungin |
| --- | --- | --- | --- | --- | --- | --- | --- | --- |
| Species | Nursing home | MIC | Resistance (MIC > 1) | MIC | Resistance (MIC > 1) | MIC | Resistance (MIC > 1) | MEC |
| *Aspergillus fumigatus* | A | 1 | S | 1 | S | 1 | S | 0.5 |
| *Aspergillus fumigatus* | A | 1 | S | 1 | S | 1 | S | 0.5 |
| *Aspergillus fumigatus* | A | 0.25 | S | 0.5 | S | 0.5 | S | 0.5 |
| *Aspergillus fumigatus* | A | 0.25 | S | 1 | S | 0.5 | S | 0.25 |
| *Aspergillus fumigatus* | A | 0.25 | S | 0.25 | S | 0.5 | S | 0.25 |
| *Aspergillus fumigatus* | A | 0.25 | S | 0.5 | S | 0.5 | S | 0.25 |
| *Aspergillus fumigatus* | A | 0.25 | S | 0.25 | S | 0.25 | S | 0.5 |
| *Aspergillus fumigatus* | B | > 8 | R | 2 | R | 0.5 | S | 0.25 |
| *Aspergillus fumigatus* | B | 1 | S | 1 | S | 1 | S | 1 |
| *Aspergillus fumigatus* | B | 0.5 | S | 0.5 | S | 0.5 | S | 0.5 |
| *Aspergillus fumigatus* | B | 0.25 | S | 0.5 | S | 0.5 | S | 0.25 |
| *Aspergillus fumigatus* | B | 0.25 | S | 0.25 | S | 0.25 | S | 0.5 |
| *Aspergillus fumigatus* | B | 0.25 | S | 0.25 | S | 0.25 | S | 0.5 |
| *Aspergillus fumigatus* | B | 0.125 | S | 0.25 | S | 0.5 | S | 0.25 |
| *Aspergillus fumigatus* | B | 0.125 | S | 0.25 | S | 0.25 | S | 0.5 |
| *Aspergillus fumigatus* | D | 1 | S | 1 | S | 1 | S | 0.5 |
| *Aspergillus fumigatus* | D | 0.5 | S | 0.5 | S | 0.5 | S | 0.5 |
| *Aspergillus fumigatus* | D | 0.25 | S | 0.25 | S | 0.25 | S | 0.5 |
| *Aspergillus fumigatus* | E | 0.5 | S | 0.5 | S | 2 | R | 0.25 |
| *Aspergillus fumigatus* | E | 0.5 | S | 1 | S | 1 | S | 0.5 |
| *Aspergillus fumigatus* | E | 0.5 | S | 0.5 | S | 1 | S | 0.25 |
| *Aspergillus fumigatus* | E | 0.5 | S | 0.5 | S | 1 | S | 0.25 |
| *Aspergillus fumigatus* | E | 0.5 | S | 0.25 | S | 1 | S | 0.5 |
| *Aspergillus fumigatus* | E | 0.25 | S | 0.5 | S | 0.5 | S | 0.125 |
| *Aspergillus fumigatus* | E | 0.25 | S | 0.5 | S | 0.5 | S | 0.25 |
| *Aspergillus fumigatus* | E | 0.25 | S | 0.25 | S | 0.5 | S | 0.5 |
| *Aspergillus fumigatus* | E | 0.25 | S | 0.5 | S | 0.5 | S | 0.5 |
| *Aspergillus fumigatus* | E | 0.25 | S | 0.5 | S | 0.5 | S | 0.5 |
| *Aspergillus fumigatus* | E | 0.25 | S | 0.5 | S | 0.5 | S | 0.5 |
| *Aspergillus fumigatus* | E | 0.25 | S | 0.25 | S | 0.25 | S | 0.5 |
| *Aspergillus fumigatus* | E | 0.25 | S | 0.25 | S | 0.25 | S | 0.5 |
| *Aspergillus fumigatus* | E | 0.25 | S | 0.25 | S | 0.25 | S | 0.25 |
| *Aspergillus fumigatus* | E | 0.25 | S | 0.25 | S | 0.25 | S | 0.5 |
| *Aspergillus fumigatus* | E | 0.25 | S | 0.25 | S | 0.25 | S | 0.5 |
| *Aspergillus fumigatus* | E | 0.125 | S | 0.25 | S | 0.25 | S | 0.5 |
| *Aspergillus fumigatus* | E | 0.125 | S | 0.25 | S | 0.25 | S | 0.5 |
| *Aspergillus fumigatus* | E | 0.125 | S | 0.5 | S | 0.25 | S | 0.5 |
| *Aspergillus fumigatus* | E | 0.125 | S | 0.25 | S | 0.25 | S | 0.5 |
| *Aspergillus fumigatus* | E | 0.125 | S | 0.25 | S | 0.125 | S | 0.125 |
| *Aspergillus fumigatus* | E | 0.0625 | S | 0.25 | S | 0.5 | S | 0.25 |
